# Agents to reduce cytokine storm

**DOI:** 10.12688/f1000research.9092.1

**Published:** 2016-12-22

**Authors:** Herwig Gerlach

**Affiliations:** 1 Department of Anesthesia, Critical Care Medicine, and Pain Management, Vivantes – Klinikum Neukoelln, Klinik fuer Anaesthesie, operative Intensivmedizin und Schmerztherapie, Berlin, Germany

**Keywords:** cytokine storm, sepsis, septic shock, hyperinflammation

## Abstract

The increasing insight into pathomechanisms of dysregulated host response in several inflammatory diseases led to the implementation of the term “cytokine storm” in the literature more than 20 years ago. Direct toxic effects as well as indirect immunomodulatory mechanisms during cytokine storm have been described and were the basis for the rationale to use several substances and devices in life-threatening infections and hyperinflammatory states. Clinical trials have been performed, most of them in the form of minor, investigator-initiated protocols; major clinical trials focused mostly on sepsis and septic shock. The following review tries to summarize the background, pathophysiology, and results of clinical investigations that had implications for the development of therapeutic strategies and international guidelines for the management of hyperinflammation during syndromes of cytokine storm in adult patients, predominantly in septic shock.

## Introduction

Whereas a localized and controlled inflammatory reaction helps to control inflammation and infection, a dysregulated response with subsequent hyperinflammation and cytokine storm may lead to multiple organ failure and determines the course and prognosis of the patient’s condition. This problem is apparent in different fields of medicine, one of which is the area of critical infections and septic shock. Hence, knowing the options and limitations of agents and devices to inhibit cytokine storm may be life-saving.

Unfortunately, some clinical cases, such as the desperate self-administration of high-dose endotoxin to fight a newly diagnosed cancer by a lab technician ^1^ or the phase I trial with a receptor antagonist in healthy volunteers ^2^ aiming at stimulation of host response, ended up with an overwhelming cytokine storm leading to dramatic clinical courses but provided important insight into the pathomechanisms of cytokine regulation. Adequate feedback inhibition at the cellular level seems to be a key element of regulated physiologic host response, and any attempt to bypass these mechanisms should be tested thoroughly before applying them to patients.

In the following, some background information is provided to give a brief historical overview of the medical fields where cytokine storm may play a role, of basic pathophysiology and reasons for dysregulation, and of clinically tested agents and devices, which were used to control hyperinflammation, mainly in the field of infections and sepsis. It will be demonstrated that, at present, most of these approaches are a far way from being clinically routine and remain experimental.

## Background

The first citation for the term “cytokine storm” in standard medical literature is found in 1993 in an article by Ferrara *et al*. on the effect of interleukin-1 (IL-1) on graft-versus-host disease (GVHD) after transplantation ^3^. In the following years, GVHD remained a main area for this term, and, in 1996, Aikawa was the first author to describe cytokine storm as the key pathway to multiple organ dysfunction syndrome (MODS) after surgical infections and sepsis ^4^. In 1991, a similar term, “cytokine release syndrome” (CRS), was created by Alegre *et al*. in experimental models of hyperinflammation ^5^; in hematologic malignancies, the use of modern approaches like infusion of “chimeric antigen receptor” (CAR) T cells may lead to severe toxicities such as CRS ^6^. The clinical course is often similar to that of septic hyperinflammation with subsequent MODS, thus requiring interventions as used for cytokine storm in infections ^6^.

Several hundred articles have since followed, and other clinical syndromes were brought into the context of cytokine storm and CRS, such as different viral diseases (especially influenza infection), bacterial infections, hemophagocytic lymphohistiocytosis, multiple sclerosis, pancreatitis, and other inflammatory diseases leading to MODS ^7, 8^. The specific effect of some bacterial toxins on host response regulation inducing cytokine storm with subsequent septic shock is the reason to take this field of critical care medicine as an example to clarify pathophysiology and therapeutic approaches ^9^.

Sepsis is a complex and life-threatening syndrome induced by a dysregulated host response to infections ^10^. During local infections, a physiologic inflammatory response helps to control the focus, whereas a dysregulated host response leads to macro- and micro-circulatory failure, thus inducing organ dysfunction, which determines the patient’s symptoms and the clinical course of disease ^10, 11^. For administrative documentation in the daily clinical practice of intensivists, different morbidities are often ascribed, although patients finally die from the sequelae of sepsis, which makes it difficult to reliably generate epidemiologic data from available intra-hospital data files. Thus, outcome data often result from prospective regional cohorts ^12^; recent large studies tried to describe epidemiology on a multi-national level ^13^. The most affected organs by sepsis and septic shock are the lungs, the cardiovascular system, and the kidneys ^14^. With an estimated mortality rate of 40% to 60%, septic shock is the focus of adult critical care medicine, and implementation of evidence-based methods and individual, goal-oriented strategies are the key approaches against this increasingly prevalent and life-threatening disease.

The inflammatory processes, which play a role in the pathogenesis of diseases like septic shock or other hyperinflammatory states, have certain similarities. They represent a physiologic host response by the immune system against endogenous (for example, tissue necrosis) or exogenous (for example, microorganisms and trauma) stimuli to protect the organism and to restore homeostasis ^15^. Hence, inflammation is an essential part of the innate as well as the adaptive immune system. In the initial phase, the inflammation is often a predominantly local syndrome with a more or less pronounced transient systemic response. On the other hand, this systemic inflammatory response syndrome (SIRS) is potentially harmful when it is part of a generally overwhelming process. This may lead to circulatory instability by vasodilation due to the production of nitric oxide and to ongoing microcirculatory failure ending with a single or combined organ dysfunction or failure (MODS) ^16^. Although the concept of SIRS as a key element of the pathophysiology of sepsis has been used for more than two decades, it was omitted from the recently published, new definitions of sepsis ^10^ since there are limitations to how the different courses of the disease can be explained. The question of whether “SIRS” as a clinical entity should be kept for defining or (just) declaring sepsis is part of a broad discussion in the current literature.

The control of local and systemic pro-inflammatory mechanisms by anti-inflammatory counterbalance is an important protective process against further enhancement of inflammation. If, however, the anti-inflammatory reaction gets too strong, this may lead to decreased immune competency with so-called “second hit” infections (for example, after major surgery) ^17^. Thus, the local and systemic imbalance between pro- and anti-inflammation is a crucial aspect of the pathogenesis of systemic inflammatory response and multiple organ dysfunctions ^18^. This is especially important for patients with sepsis, after multiple trauma, or after major surgery, who are often in an immunosuppressive phase and not only in a phase of uncontrolled hyperinflammation. Components taking part in these pro- and anti-inflammatory processes are found in the innate immune system, mainly as endothelial cells, polymorphonuclear cells, macrophages, and so on, as well as in the adaptive immune system, represented by specific humoral B-cell and cellular T-cell immunity ^19^. Additional components are the coagulation as well as the complement system, eicosanoid metabolism, and the endocrine system.

## Dysregulation of host response leading to cytokine storm

**Figure 1.**
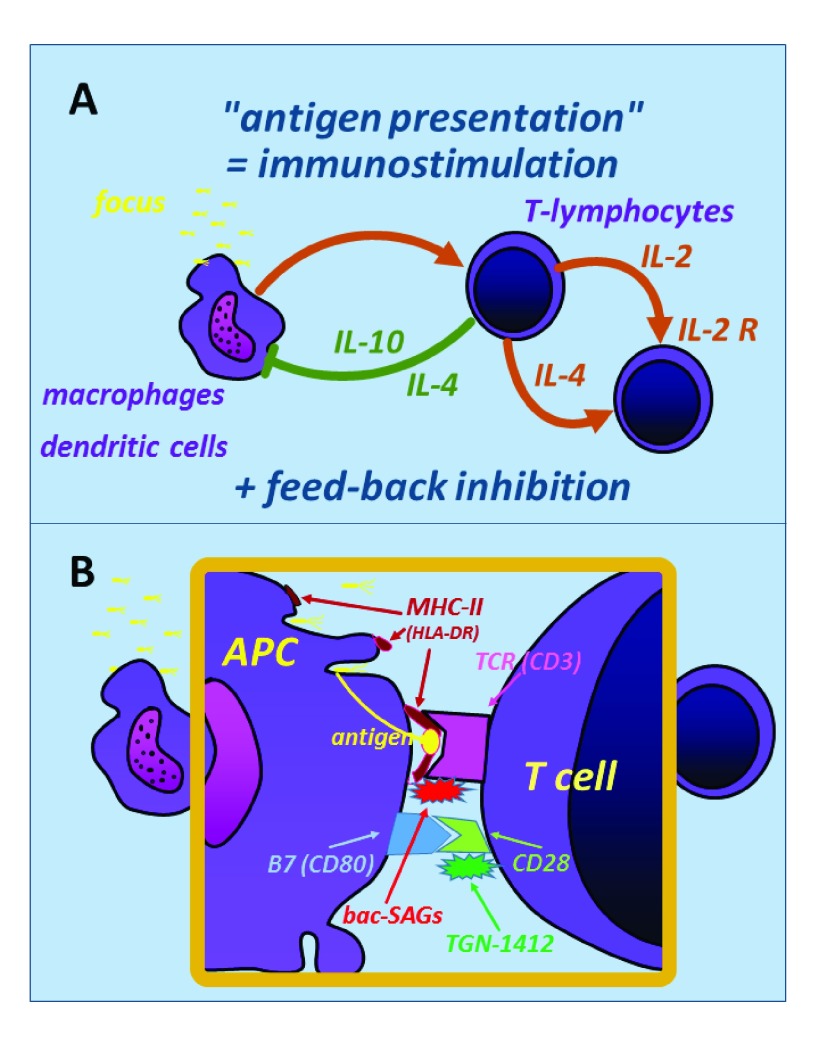
Simplified scheme of the regulatory interaction between the antigen-presenting cell (APC) (for example, macrophages and dendritic cells) from the innate immune system (left) and the naïve T cell from the adaptive immune system (right). ( **A**) In general, antigen presentation induces both immunostimulatory and anti-inflammatory pathways (feedback inhibition), for example, by expression of interleukin-4 (IL-4) and IL-10, which inhibits further inflammatory responses by macrophages. ( **B**) The APC–T-cell interaction involves several receptor systems: major histocompatibility complex type II (MHC-II) and T-cell receptor interact for direct antigen presentation. Bacterial superantigens (bac-SAGs) are able to “fool” this system by bypassing the inhibitory pathways, thus inducing hyperinflammation and cytokine storm. B7-CD28 receptors are also important for regulatory immunomodulation; application of the agonistic anti-CD28 antibody (TGN-1412) in humans induced a CD28 crosslinking and unrestrained hyperstimulation of T cells, which resulted in dramatic clinical courses during the initial phase I trial in healthy volunteers (“TeGenero catastrophe” in 2006 ^2^).

In general, the balance of pro- and anti-inflammatory mechanisms is warranted by a close interaction between the innate immune system represented by the group of so-called “antigen-presenting cells” (APCs) with the adaptive immune system in the form of naïve T cells. After macrophagocytosis, the APCs take fragments of microbial toxins or proteins (or both) and express these via their major histocompatibility complex type II (MHC-II) receptors, binding to the corresponding T-cell receptor (TCR) ( Figure 1). In a complex downstream pathway, the responding cells of the adaptive immune system exert both pro- and anti-inflammatory mechanisms, the latter being an important feedback inhibition (for example, via IL-4 and IL-10) to downregulate the pro-inflammatory activities of the innate immune system ( Figure 1A).

At the molecular level, several receptor systems are involved to keep the regulation of the host response balanced ( Figure 1B). If these systems are disturbed, extreme hyperinflammation may occur: this is typical for toxic shock syndrome, where bacterial “superantigens” (for example, Staphylococcal enterotoxin B) are able to “fool” the MHC-II–TCR system ( Figure 1B, upper part). The inhibitory downstream pathways are bypassed, and specific kinase systems within the T cells are activated, thus inducing a fast and overwhelming cytokine storm ^20^. A similar effect is initiated in humans by the application of an agonistic anti-CD28 antibody, which was previously tested in macaques without side effects ^2^. However, even the small difference in the genetic sequence of the CD28 receptor molecule between macaques and humans was enough to change the binding site. This induced a crosslinking between CD28 receptors with an unrestrained hyperinflammation; the first clinical symptoms in healthy volunteers appeared within minutes after intravenous application, and, a few hours later, multiple organ dysfunction occurred in all healthy volunteers ^2^.

## Clinical approaches to fight cytokine storm

As the mechanisms of cytokine storm are becoming better defined, interventions aiming to interfere with the host response have been undertaken, largely with disappointing results. Moreover, it was concluded that immunomodulating approaches in patients with sepsis, known collectively as “adjunctive therapy”, have to orientate in the patient’s immunologic competence and inflammatory as well as infectious status. Besides low-dose hydrocortisone and activated protein C, which have been demonstrated to disrupt dysfunctional cascades, thus favorably influencing the course of the disease, the use of intravenous immunoglobulins (ivIGs) has been implemented as part of adjunctive therapy. In experimental studies, these strategies have helped to reduce the incidence of infections, support failing organs, and prevent complications.

## Intravenous immunoglobulins

In 1981, Imbach *et al*. were the first clinicians infusing high-dose ivIGs in children with idiopathic thrombocytopenic purpura (that is, in an autoimmune disease) ^21^. In later years, ivIG therapy was established in many diseases with some kind of hyperinflammatory state, some of which had the highest grade of recommendation as first-choice therapy, such as Guillain-Barré syndrome, chronic inflammatory demyelinating polyradiculoneuropathy, multifocal motor neuropathy, Kawasaki vasculitis, myasthenia gravis, multiple sclerosis, antineutrophil cytoplasmatic autoantibody-positive vasculitis, and corticosteroid-resistant dermatomyositis ^22^.

On first view, the immunomodulatory pathways of administered ivIGs in hyperinflammatory diseases are difficult to understand. The “Janus-like” character of immunoglobulins can be explained by their molecular structure: immunoglobulin molecules have a variable *antigen-binding fragment* ( *Fab region*), which binds specifically to *epitopes* such as toxins or microbial surface antigens, thus defining the *idiotype* of the molecule. By using the enzyme papain, the Fab region of the molecule can be split from the rest of the molecule, which crystallizes after this fragmentation and thus is called *crystallizable fragment* (Fc region). This part is relatively constant and has the capability to bind to different subclasses of *Fc receptors* ( *type I*, *II*, and *III*), exerting immunomodulatory effects by activating or inhibiting mechanisms (or both) of many cell types. By these two molecular pathways, ivIGs can exert direct anti-toxic effects, as well as pro- and anti-inflammatory activities ^23, 24^. The latter is partially dependent on its concentration; the pathways include both the Fab region, by direct binding to surface antigens of endogenous cells such as granulocytes and T cells, and the Fc region ^24^. Specific activating or inhibitory receptors binding to the Fc region of immunoglobulins, abbreviated as FcγRs, are expressed by many cells of the innate and adaptive immune system. Activating FcγRs are found on natural killer cells, monocytes, macrophages, and dendritic cells as well as on neutrophils and B cells. Inhibitory FcγRs are expressed by B cells, monocytes, and macrophages.

Inhibitory FcγRs can further decrease the production and expression of pro-inflammatory cytokines; for example, anti-inflammatory cytokines such as IL-10 were shown to be expressed during viral encephalitis with a hyperinflammatory state by inhibitory FcγRs on regulatory T cells, thus exerting a strong anti-inflammatory and immunosuppressive effect ^25^. Moreover, it was shown that in different diseases, the complement system is influenced by inhibitory ivIGs ^26^. Low-dose ivIGs probably require complement activation or binding of the Fc fragment to FcγRs on innate immune effector cells ^23^, whereas high doses of ivIGs are more likely to act via the direct pathways with immune cells. In conclusion, the complex mechanisms by which immunoglobulins exert both anti-toxic and immunomodulatory effects are beginning to be understood, and some of the findings mentioned here may have important implications for the future use of ivIGs in different diseases, including infections, septic shock, and hyperinflammatory states ^27, 28^.

## Intravenous immunoglobulins in toxic shock syndrome

Streptococcal toxic shock syndrome (STSS) is the most severe manifestation of invasive infections based on *Group A Streptococcus*. There is increasing evidence that the clinical course results from excessive cytokine production induced by bacterial exotoxins belonging to the family of Gram-positive bacterial superantigens. ivIGs are able to block T-cell activation by the aforementioned Fc-dependent anti-inflammatory mechanisms; in addition, ivIGs contain superantigen-neutralizing antibodies, thus exerting a direct anti-toxic effect. Some clinical data are from children ^29^; after several minor observational trials in adults had encouraging results ^30^, a European, randomized, double-blind, placebo-controlled trial was initiated but had to be stopped because of low inclusion rate of patients ^31^. Although the final number of patients was very low, there was still a more-than-threefold increased mortality in the placebo group ^31^. Together with the recent data in children ^29^, these data give support for a weak suggestion to consider ivIGs in patients with STSS, although sufficient evidence is still missing ^32^.

## Specific antibodies against toxins and cytokines

A source of infection may result in the release of bacterial toxins like components of the cell wall into the bloodstream, and these toxins interact with the cells of the immune system, causing the release of endogenous mediators such as tumor necrosis factor (TNF) or IL-1, thus causing cardiovascular insufficiency, hypotension, and decreased end-organ perfusion. The first large randomized controlled trial (RCT) using a monoclonal antibody against the Lipid A fraction of Gram-negative endotoxin, however, was disappointing ^33^, and it was concluded that more investigation is required before these drugs can be used in patients having or suspected of having Gram-negative sepsis. Later, human monoclonal antibodies against specific antigens of bacteria were also tested but did not result in any benefit for the patients ^34^; altogether, the current view on the development of specific monoclonal antibodies against bacterial antigens for the treatment of sepsis is rather skeptical.

In parallel with these trends for specific antigen blockade, it was speculated that it may be useful to inhibit the endogenous cytokines with anti-TNF or anti-IL-1 preparations as part of a complex adjunctive therapy strategy of sepsis and multiple organ failure ^35, 36^. For TNF, the strategy was followed, and a specific antibody was developed; although initial experimental data were quite positive, a large international RCT failed to show efficacy ^37^. For IL-1, another way to block this cytokine was chosen, using an endogenous IL-1 receptor antagonist. Like the anti-TNF trial, a large RCT did not result in a reduced mortality in treated patients ^38^. However, a recent *post hoc* analysis of the anti-IL-1 trial supports the concept of defined entities within the heterogeneous group of septic patients who might benefit from cytokine neutralization, thus supporting the principle of a more “individualized” approach for using neutralizing antibodies ^39^.

In conclusion, neither direct antibodies against bacterial toxins or surface antigens (or both) nor anti-cytokine preparations could show a positive effect in adult patients with cytokine storm during sepsis or septic shock. Possibly, STSS is different owing to its specific pathogenesis. Although these data are rather frustrating, investigators are still convinced that future research should concentrate on the clarification of immunologic pathways of sepsis with subsequent organ failure in order to develop innovative strategies against this life-threatening disease ^40, 41^.

## Other options to reduce cytokine storm

There are numerous experimental approaches to block cytokine storm; most of them try to interfere with upstream mechanisms (that is, reduce the synthesis of cytokines). However, no specific therapies are available at the moment. One very “old” approach is the application of low-dose steroids, although there is no evidence that, especially during cytokine storm, these doses are enough to inhibit the pathomechanisms adequately. It is more a supportive measure to stabilize hemodynamics and, for this reason, is still part of the current international guidelines, but with a low grade of evidence ^42^.

In hematologic malignancies, CRSs during CAR T-cell therapy may be treated with specific anti-IL-6 antibodies (tocilizumab), which are available for other diseases like arthritis and vasculitis ^43^. There is no clear evidence by larger clinical trials, and tocilizumab is still not approved for this indication; however, off-label use of tocilizumab is an option to reduce CRS-induced organ failure, although there are some concerns that the benefit of CAR T-cell therapy may be reduced ^6^. Another approach is the use of high-dose corticosteroids and this once again demonstrates the similarities between infectious hyperinflammation and CRS during therapy of hematologic malignancies.

A recent approach is to absorb cytokines with special cartridges, which are part of extracorporeal circulation devices (hemofiltration). There are some promising experimental data ^44^, and the first case reports in clinical use have been reported ^45^. A more indirect approach is to block the complement cascade via inhibition of the C5a pathway, but reliable clinical data for this approach are still missing ^46^. Moreover, mesenchymal stem cells (MSCs) were tested, and there is some indirect evidence (for example, improved bacterial clearance) that MSC transplantation may be beneficial; so far, however, no large clinical trial supports the routine use of this method, especially not in the subgroup of patients with diagnosed cytokine storm or CRS ^47^. Finally, several anti-cancer drugs have been tested with the idea that their blocking activities in cell replication may be beneficial in states of overwhelming immune response like septic shock; once again, the present evidence is not enough to give any recommendations for routine clinical use ^48^.

This short list is a far way from being complete, and there are many other experimental approaches to inhibit hyperinflammation. However, no other so-called “adjunctive therapy” could reveal sufficient clinical evidence, such as glycemic control, selenase, specific antibodies, alkaline phosphatase, thiamine, Toll-like receptor inhibitors, nitric oxide inhibitors, glutamine, lactoferrin, statins, and many more, which were all tested in clinical trials but failed to provide any benefit ^40, 41^.

## Closing remarks

In adult patients with infections, sepsis, or septic shock, there is currently no high-grade evidence for any approach to block cytokine storm owing to lack of sufficient data; concerning the unclear risk-benefit ratio and high costs of some of these approaches, a strong recommendation cannot be given. This is frustrating given the quite-convincing progress in basic research, which has resulted in a growing understanding of the pathomechanisms leading to overwhelming clinical syndromes of cytokine storm. Only for the treatment of STSS, the combined anti-toxic and immunomodulatory effect of ivIGs may be of some advantage, especially in children. The TGN-1412 catastrophe demonstrated that, although the preclinical development followed established regulations, even small differences between animal and human genetic information will remain an incalculable risk. Recent trials with a more “smooth” absorption of extreme cytokine levels without interfering with the upstream synthesis regulation may be a reasonable alternative to single-hit specific inhibitors, which all keep the risk of a further dysbalance of the immunomodulatory system. This lack of a “magic bullet” against syndromes of cytokine storm was disappointing in the past; however, it also reminded us to keep focused on the patients’ clinical state with all options of supportive measures in critical care that are available at the moment—and this armament is not too bad.
